# Effects of single-nucleotide polymorphisms in the mTORC1 pathway on the risk of brain metastasis in patients with non-small cell lung cancer

**DOI:** 10.1007/s00432-019-03059-y

**Published:** 2019-10-22

**Authors:** Yiquan Xu, Yina Huang, Lihong Weng, Jiankun Zheng, Yi Huang, Ying Lin, Yunan Zhao, Hongru Li, Yusheng Chen

**Affiliations:** 1 Shengli Clinical Medical College of Fujian Medical University, Fuzhou, China; 2 Department of Respiratory Medicine and Critical Care Medicine, Fujian Provincial Hospital, No. 134 East Street, Fuzhou, 350001 China

**Keywords:** mTORC1, Genetic polymorphisms, Brain metastasis, Risk, Biomarker

## Abstract

**Purpose:**

The mammalian target of rapamycin complex 1 (mTORC1) signaling pathway plays a vital role in cancer development and progression. This study aimed to investigate the relationship between genotype variants in mTORC1 pathway and the risk of brain metastasis (BM) in patients with non-small cell lung cancer (NSCLC).

**Methods:**

We extracted genomic DNA from blood samples of 501 NSCLC patients and genotyped eight single-nucleotide polymorphisms (SNPs) in three core genes [mammalian target of rapamycin (mTOR), mammalian lethal with sec-13 protein 8 (mLST8) and regulatory-associated protein of mTOR (RPTOR)] of the mTORC1 pathway. The associations between these SNPs and the risk of BM development were assessed.

**Results:**

The AG/GG genotype of mLST8:rs26865 and TC/CC genotype of mLST8:rs3160 were associated with an increased risk of BM [hazard ratios (HR) 2.938, 95% confidence interval (CI) 1.664–5.189, *p *< 0.001 and HR = 2.490, 95% CI = 1.543–4.016, *p *< 0.001, respectively]. These risk polymorphisms had a cumulative effect on BM risk, with two risk genotypes exhibiting the highest increased risk (*p *< 0.001). Furthermore, these risk SNPs were associated with the lymph node metastasis (N2/3), body mass index (BMI) (≥ 25 kg/m^2^), high level of squamous cell carcinoma (SCC) antigen and Ki-67 proliferation index. Moreover, patients with AG/GG genotype of mLST8:rs26865 had significantly lower median overall survival than those with AA genotype (12.1 months versus 21.6 months, *p *= 0.04).

**Conclusions:**

Our results indicate that polymorphisms in mTORC1 pathway were significantly associated with increased risk of BM and may be valuable biomarkers to identify NSCLC patients with a high risk of BM.

**Electronic supplementary material:**

The online version of this article (10.1007/s00432-019-03059-y) contains supplementary material, which is available to authorized users.

## Introduction

Primary lung cancer is among the most common malignant tumors with the highest mortality worldwide (Chen et al. 2016; Siegel et al. 2016). Non-small cell lung cancer (NSCLC) accounts for almost 80% of all lung cancer cases (Hoffman et al. 2000) and has the highest incidence of brain metastasis (BM) (Nguyen et al. 2009). Approximately, 20–65% of patients with NSCLC will have BM during the course of the disease and ultimately die from it (Chen et al. 2014; Olmez et al. 2010; Preusser et al. 2012). Despite great progress in systemic therapy including surgery, radiotherapy, chemotherapy, and targeted therapy, the prognosis of BM patients remains generally poor, with a median survival time of approximately 13.7 months (Baek et al. 2018). Without any treatments, this further decreases to approximately less than 2 months (Chen et al. 2017). BM has become an important cause of lung cancer morbidity and mortality (Nayak et al. 2012). Therefore, identifying predictive factors of BM is essential for its prevention and improving patient survival.

Some previous studies have reported higher incidence of BM in those who are younger, female, have stage IIIB/IV disease, non-smokers, and with EGFR mutation (Hsiao et al. 2013; Wei et al. 2018). Other studies have also suggested that the size of the primary tumor, cell type, and intrathoracic lymph node stage were associated with BM (Mujoomdar et al. 2007). However, these studies did not take genetic factors and molecular mechanisms into account. BM develops via a complicated process that encompasses lung cancer cell invasion, migration, arrest, extravasation, and formation of micro-metastases (Hanahan and Weinberg 2011; Paduch 2016). It has been reported that matrix metalloproteinase (MMP) overexpression promotes the transmigration of lung cancer cells to the brain parenchyma through the blood vasculature and enhance the risk of BM (Feng et al. 2011; Hu et al. 2010). In addition, the expression of chemotactic factors including CXCL12 and its receptor CXCR4 was significantly higher in BM than in primary lung cancer; CXCR4 may improve the adhesion and chemoresistance of lung cancer cells, leading to BM (Chen et al. 2011; Paratore et al. 2011).One study of mice has shown that WNT/TCF signaling plays an important role in the formation of brain and bone metastasis through LEF1 and HOXB9 (Nguyen et al. 2009). Recently, Aljohani et al. revealed that the Keap1-Nrf2-ARE pathway mutations facilitate cancer cell migration to the distant sites in patients with BM (Aljohani et al. 2018). Despite extensive studies on the key molecular mediator of BM, its incidence rate has not markedly increased, highlighting the importance of identifying novel genetic biomarkers for BM.

Single-nucleotide polymorphisms are among the most common genetic variants and play an important role in the development, invasion, and prognosis of lung cancer. Therefore, SNPs may be a valuable molecular marker for assessing the risk of BM. Some studies have demonstrated that genetic variants of the phosphoinositide 3-kinase-Akt pathway and autophagy-related gene ATG16L1 are related to an increased risk of BM in patients with NSCLC (Li et al. 2013b, 2017). In this study, we focus on the relationship between genetic variation in mTORC1 pathway and the risk of BM patients in NSCLC.

In mammalian cells, mTOR signaling pathway has two forms: mTORC1 and the mTORC2. Of these, mTORC1 is composed of mTOR, mLST8, and RPTOR (Yang and Guan 2007). It has been proven to be a central regulator of numerous cellular processes, including sensing nutrient signaling, controlling translation and protein transport of mRNA in cells, and protein degradation (Sarbassov et al. 2005). More importantly, it is closely related to the occurrence and development of cancers (Dazert and Hall 2011). Previous studies have confirmed that alterations in the mTORC1 complex are associated with the occurrence of several cancers, including prostate cancer, liver cancer, and colon cancer and are sensitive to rapamycin inhibition (Chen et al. 2015; Kaibori et al. 2015; Shuhua et al. 2015). Another study revealed that functional SNPs of the mTORC1 gene may increase the risk of esophageal squamous cell carcinoma individually or collectively (Zhu et al. 2013). In addition, two similar studies have confirmed that mTORC1 polymorphisms are associated with the prognosis of gastric cancer (GC), and the risk of death increased more than twofold when GC patients have three risk genotypes of the mTORC1 SNPs (Xue et al. 2018). Collectively, these studies suggest that mTORC1 polymorphisms may be important biomarkers of cancer development, progression, and prognosis. However, no study has evaluated the potential role of functional SNPs in the susceptibility to BM from NSCLC.

Thus, this study aimed to investigate the association between eight potential functional polymorphisms of three genes (i.e., mTOR, mLST8, and RPTOR) in the mTORC1 pathways and the risk of BM in patients with NSCLC in the Chinese population. Moreover, given that BM of NSCLC is a polygenic disease, we also aimed to investigate the effect of combination of multiple SNPs on the prognosis of BM and the relationship between risk genotypes and various tumor-related indicators including serum tumor makers, Ki-67 proliferation index, and gene mutation frequency of BM from NSCLC.

## Materials and methods

### Study population and clinical data collection

This retrospective analysis was approved by the Medical Ethics Committee of Fujian Provincial Hospital. We evaluated 540 patients from Fujian Provincial Hospital (Fujian, China) between May 2015 and October 2017. All patients were newly diagnosed with NSCLC confirmed via histopathological examinations. There were no limitations on age, sex, histology, or disease stage, but sufficient DNA of blood samples for genotype analysis was required. The exclusion criteria were having other cancers and tumors of an unknown origin. Demographic data, including age, sex, ethnicity, body mass index (BMI), smoking status, occupational exposures to potential carcinogens, medical history, and family history of cancer, were collected using a structured questionnaire administered during individual interviews. At the end of the interview, each patient signed a written informed consent for blood sample collection for scientific research and donated 3 ml peripheral blood for genomic DNA extraction.

The patients’ clinicopathological data, including disease stage, depth of invasion, lymph node and metastasis status, treatment regimens, and gene mutation status, were obtained from medical records. Pretreatment imaging findings and serum tumor markers were assessed and used for analysis. NSCLC staging was according to the tumor–node–metastasis criteria of the American Joint Commission on Cancer (AJCC) in 2017 (the 8th edition). The diagnosis of BM was based on computed tomography scans or magnetic resonance imaging scans.

We followed up all patients through outpatient service and telephone calls every 2–3 months. BM and survival information was obtained from follow-up records of each patient. The last follow-up was in July 2019.

The time to BM was defined as the duration between the date of NSCLC diagnosis and the date of BM diagnosis. Patients without BM were censored at the date of last contact. The survival time of BM with NSCLC was defined as the time between the date of BM diagnosis and the date of cancer-related death. Progression-free survival was calculated from the date of BM diagnosis to the date of disease progression.

Of all patients included in this study, 39 were excluded, 19 because of incomplete data on disease staging and 20 lost to obtained information on BM, leaving 501 patients with complete information for this analysis.

### Polymorphism selection

To choose the common and potentially functional SNPs in three core genes of the mTORC1 signaling pathway (mTOR, mLST8, and RPTOR) in the Chinese Han population, we used the public HapMap SNP database (http://www.ncbi.nlm.nih.gov/) and SNPinfo (http://snpinfo.niehs.nih.gov/). The selection criteria were as follows: minor allele frequency ≥ 5%, linkage disequilibrium coefficient *r*^2^ < 0.8, located in the regulatory region of genes, and affecting the function of transcription factor binding site or the microRNA binding site.

### Genomic DNA isolation and genotype analysis

Genomic DNA was extracted from peripheral blood using an EasyPure Blood Genomic DNA kit (EE121, TransGen Biotech) according to the manufacturer’s protocols and stored at − 80 °C until use. For genotyping, we designed primers for eight SNPs (Supplemental Table S1) and used the matrix-assisted laser desorption/ionization-time-of-flight mass spectrophotometry (MALDI–TOF-MS) to detect allele-specific primer extension products, and the MassARRAY platform (Agena Bioscience) and Sequenom TYPER software (version 4.1) were used to analyze the assay data. To evaluate data reproducibility, we performed a blind, randomized, repeated analysis of 5% of the DNA samples, and the results showed a reproducibility of 99%.

### Statistical analysis

Log-rank tests were conducted to compare the difference in survival time between patients with brain metastasis and without brain metastasis. Cox proportional hazards regression analysis was used to investigate the influence of genotypes on BM risk, and the analysis was also adjusted for age, sex, smoking status, BMI, Karnofsky Performance Score (KPS), tumor histology, and disease stage. Kaplan–Meier curves were used to assess the cumulative probability of BM. The *χ*^2^ test was used to evaluate the differences in gene mutation frequency between groups. Student’s *t* test was performed to evaluate differences in serum tumor markers and Ki-67 proliferation index between two groups. All statistical analyses were performed with SPSS 20.0 software, and *p *< 0.05 was considered to be statistically significant.

## Results

### Clinical characteristics of the study population

The characteristics of the 501 NSCLC patients included in this study are shown in Table 1. In total, 309 (61.7%) and 192 (38.3%) patients were men and women, respectively. The median age was 60 years (range 25–90 years); 63.2% had a smoking history; 55.1% had KPS greater than 80; 82.6% had BMI < 25 kg/m^2^; and 73.2% had adenocarcinoma. With respect to AJCC stage at diagnosis, 43.9% had stage III or IV disease. Of the 130 patients who developed BM in this study, 31 (23.8%) had brain metastases concurrently at NSCLC diagnosis, while the 99 (76.2%) developed brain metastases at a median follow-up time of 24 months. There were 76 patients who had brain metastases only, while the remaining 54 patients had both brain and other site metastases. A total of 27 patients (5.4%) were lost to follow-up and the median time from NSCLC diagnosis to brain metastases was 10 months.

**Table 1:** Clinical characteristics of non-small cell lung cancer patients and their association with brain metastasis

Characteristic	Patients *N* = 501 (%)	Events *N* = 130 (%)	Univariate analysis		Multivariate analysis^a^	
			HR (95% CI)	*p*	HR (95% CI)	*p*
Sex						
Male	309 (61.7)	80 (25.9)	1.000		1.000	
Female	192 (38.3)	50 (26.0)	1.084 (0.760–1.547)	0.656	1.300 (0.835–2.024)	0.246
Age (years)						
< 60	238 (47.5)	60 (25.2)	1.000		1.000	
≥ 60	263 (52.5)	70 (26.6)	1.079 (0.763–1.527)	0.668	0.905 (0.631–1.299)	0.589
Median (range)	60 (25–90)					
Tobacco smoking status						
No	317 (63.2)	77 (24.3)	1.000		1.000	
Yes	184 (36.8)	53 (28.8)	0.914 (0.637–1.311)	0.625	0.990 (0.594–1.649)	0.969
KPS score						
> 80	276 (55.1)	43 (15.6)	1.000		1.000	
80	190 (37.9)	63 (33.2)	3.861 (2.312–6.447)	< 0.001	1.404 (0.812–2.430)	0.225
< 80	35 (7.0)	24 (68.6)	2.465 (1.671–3.634)	< 0.001	1.116 (0.745-1.672)	0.595
Body mass index						
< 25.0	414 (82.6)	100 (24.2)	1.000		1.000	
≥ 25.0	87 (17.4)	30 (34.5)	0.336 (0.171–0.662)	0.002	0.498 (0.246–1.005)	0.052
Tumor histology						
Squamous cell	92 (18.4)	12 (13.0)	1.000		1.000	
Adenocarcinoma	367 (73.2)	95 (25.9)	2.359 (1.228–4.533)	0.010	1.524 (0.775–2.995)	0.222
NSCLC,NOS	42 (8.4)	23 (54.8)	3.121 (1.484–6.567)	0.003	2.106 (0.981–4.522)	0.056
Disease stage at diagnosis						
I, II	281 (56.1)	53 (18.9)	1.000		1.000	
III, IV	220 (43.9)	77 (35.0)	17.581 (7.161-3.162)	< 0.001	5.148 (1.814-14.615)	0.002
Depth of invasion						
Tis, T1, T2	338 (67.4)	75 (22.2)	1.000		1.000	
T3, T4	163 (32.6)	55 (33.7)	1.920 (1.281–2.877)	0.002	0.701 (0.467–1.054)	0.088
Lymph node metastasis						
N0, N1	291 (58.0)	36 (12.4)	1.000		1.000	
N2, N3	210 (42.0)	94 (44.8)	3.563 (2.404–5.282)	< 0.001	1.586 (1.003–2.506)	0.048
Distant metastasis						
M0	303 (60.5)	62 (20.5)	1.000		1.000	
M1	198 (39.5)	68 (34.3)	23.470 (11.450–48.105)	< 0.001	0.975 (0.456–2.086)	0.949

^a^Multivariate analyses were adjusted for all the factors listed in this table

Both univariate and multivariate Cox model for analysis of the association between clinical characteristics and BM showed that the risk of BM was significantly higher in patients with stage III/IV disease (*p *= 0.002) and N2/3 lymph node metastasis (*p *= 0.048). No other characteristics were correlated with the risk of BM.

### Associations between mTOR SNPs and BM risk

**Fig. 1:**
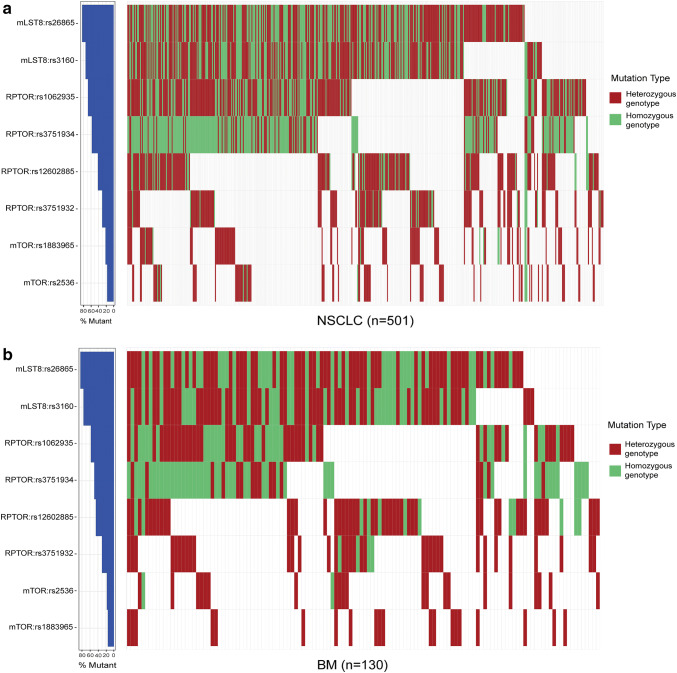
Alterations of eight candidate polymorphisms in all NSCLC patients and brain metastasis. The data was analyzed for heterozygous genotypes and homozygous genotypes. **a** Distribution of SNPs in a total of 501 non-small cell lung cancer patients. **b** Alteration of SNPs in 130 brain metastasis patients

Next, we identified a total of eight SNPs for genotyping (Supplemental Table S2): for the RPTOR gene, three SNPs in the 3′ UTR (rs3751934 C > A, rs1062935 T > C, and rs3751932 T > C) and one SNP in the 5′UTR (rs12602885 G > A); for the mTOR gene, one SNP in the intron-1 region (rs1883965 G > A) and one SNP in the 3′UTR (rs2536 T > C); for the mLST8 gene, one SNP in the promoter region (rs26865 A > G) and one SNP in the 3′UTR (rs3160 T > C). Based on the change of single locus in SNPs, we divided the SNPs into hetero- and homozygous genotypes and mapped the distribution of SNPs in NSCLC patients with or without BM (Fig. 1a, b). Of all the eight SNPs, the most highly distributed genetic polymorphisms in NSCLC was the mLST8:rs26865 AG/GG (82.4%, Fig. 1a; Table 2), followed by mLST8:rs3160 TC/CC (72.3%) and RPTOR:rs3751934 AC/CC (57.1%). Among them, mLST8:rs26865 AG/GG had the highest mutation rate in patients with brain metastases (Fig. 1b).

**Table 2:** Correlation between different genotypes of genes in MTORC1 pathway and brain metastasis

SNP’s genes	Genotypes	Patients *N* = 501 (%)	Events *N* = 130 (%)	Univariate analysis		Multivariate analysis^a^	
				HR (95% CI)	*p*	HR (95% CI)	*p*
mTOR:rs1883965	GG	394 (78.6)	110 (38.7)	1.000		1.000	
	GA + AA	107 (21.4)	20 (23.0)	0.731 (0.453–0.178)	0.198	0.755 (0.458–1.245)	0.271
mTOR:rs2536	TT	418 (83.4)	108 (34.8)	1.000		1.000	
	TC + CC	83 (16.6)	22 (36.1)	1.126 (0.716–1.770)	0.607	1.010 (0.634–1.607)	0.968
mLST8:rs26865	AA	88 (17.6)	21 (31.3)	1.000		1.000	
	AG + GG	413 (82.4)	109 (35.9)	2.444 (1.467–4.072)	0.001	2.938 (1.664–5.189)	< 0.001
mLST8:rs3160	TT	139 (27.7)	33 (31.1)	1.000		1.000	
	TC + CC	362 (72.3)	97 (36.6)	2.050 (1.316–3.195)	0.002	2.490 (1.543–4.016)	< 0.001
RPTOR:rs1062935	TT	164 (32.7)	55 (50.5)	1.000		1.000	
	TC + CC	337 (67.3)	75 (28.6)	0.678 (0.478–0.963)	0.030	0.844 (0.588–1.211)	0.357
RPTOR:rs12602885	GG	294 (58.7)	70 (31.3)	1.000		1.000	
	GA + AA	207 (41.3)	60 (40.8)	0.876 (0.615–1.247)	0.462	0.853 (0.591–1.232)	0.398
RPTOR:rs3751932	TT	349 (69.7)	90 (34.7)	1.000		1.000	
	TC + CC	152 (30.3)	40 (35.7)	0.979 (0.672–1.427)	0.913	0.900 (0.606–1.335)	0.599
RPTOR:rs3751934	CC	215 (42.9)	68 (46.3)	1.000		1.000	
	AC + AA	286 (57.1)	62 (21.7)	0.738 (0.522–1.043)	0.085	0.882 (0.616–1.264)	0.495

*HR* hazard ratio, *CI* confidence interval

^a^Multivariate analyses in this table were adjusted for age, sex, smoking status, Karnofsky Performance Status, body mass index, tumor histology, TNM stage

**Fig. 2:**
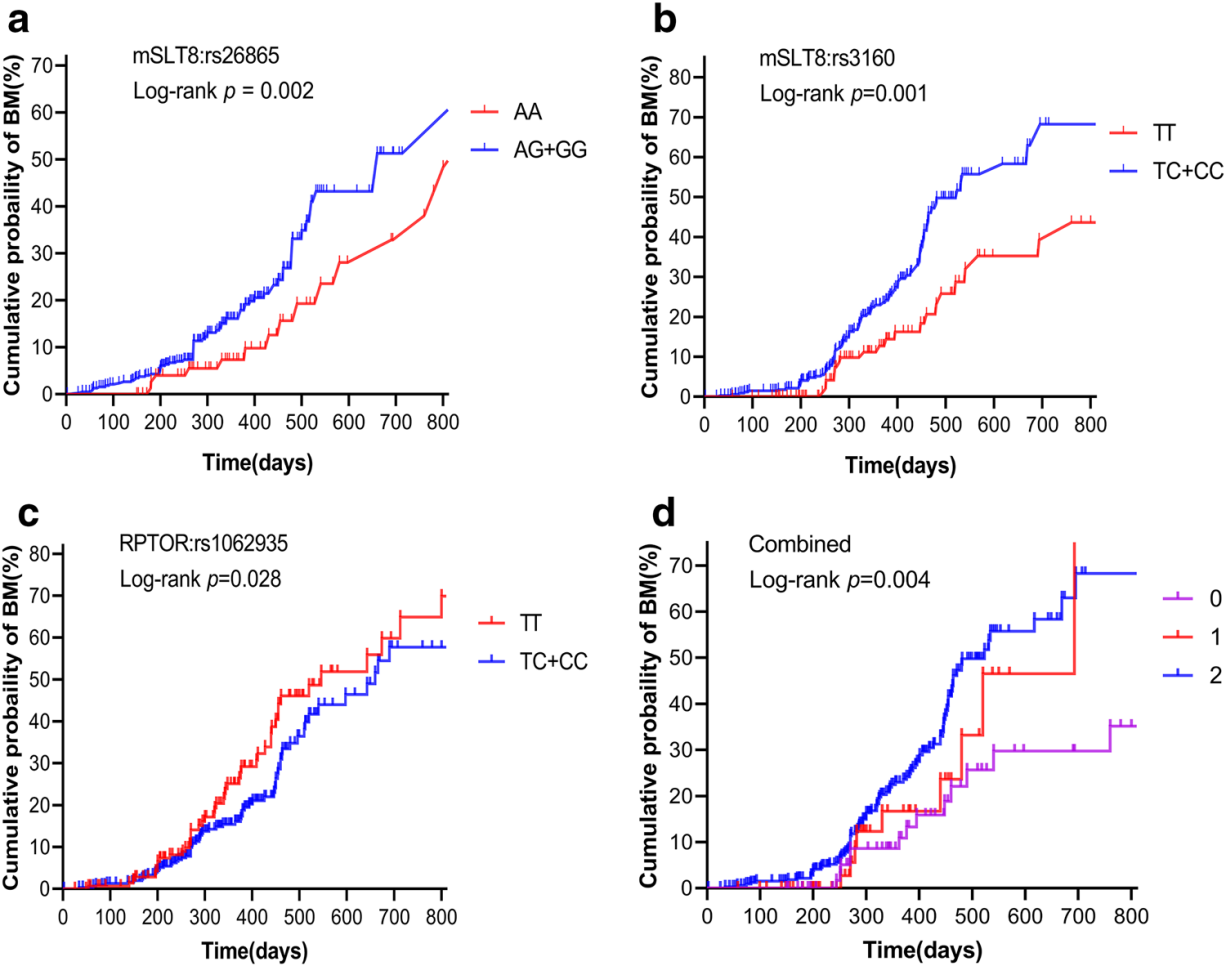
Kaplan–Meier analysis of the cumulative probability of brain metastasis in patients with non-small cell lung cancer according to the following unfavorable genotypes: **a** mLST8:rs26865, **b** mLST8:rs3160, **c** RPTOR:rs1062935, **d** combined

Our analysis of the potential correlation between each of the eight candidate SNPs and the risk of BM development using Cox model analysis revealed that mLST8:rs26865, mLST8:rs3160, and RPTOR:rs1062935 were associated with BM risk. As is shown in Fig. 2, mLST8:rs26865 with AG/GG genotype (*p *= 0.002, Fig. 2a) and mLST8:rs3160 with TC/CC genotype (*p *= 0.001, Fig. 2b) were significantly associated with increased risk of BM. By contrast, RPTOR:rs1062935 with TC/CC genotype (*p *= 0.028, Fig. 2c) was significantly correlated with a lower BM risk. These risk associations were not observed in the other five polymorphisms in mTORC1 genes.

When the distribution of risk genotype of BM was considered in the analysis, a higher ratio was found for mLST8:rs26865 with AG/GG genotype (35.9%), mLST8:rs3160 with TC/CC genotype (36.6%), and RPTOR:rs1062935 with TC/CC genotype (50.5%) in BM patients than AA (35.9%), TT (36.6%), and TC/CC (50.5%) genotype (Table 2). In addition, multivariate Cox proportional hazard model adjusted for age, sex, smoking status, KPS, BMI, tumor histology, and TNM stage to analyze the association between the eight polymorphisms and the risk of BM revealed that mLST8:rs26865 with AG/GG genotype (HR = 2.938, 95% CI = 1.664–5.189, *p *< 0.001) and mLST8:rs3160 with TC/CC genotype (HR = 2.490, 95% CI = 1.543–4.016, *p *< 0.001) were significantly associated with increased risk of BM. Meanwhile, polymorphisms of RPTOR:rs1062935 with TC/CC genotype (HR = 0.678, 95% CI = 0.478–0.963, *p *= 0.030) was significantly associated with decreased risk of BM only in univariate Cox proportional hazard analysis. Further, compared with their common genotypes, the other four SNPs showed no association with BM risk in both uni- and multivariate Cox proportional hazard analysis.

### Combined effect of polymorphisms on increased risk of brain metastasis

In the combined analysis of the effect of polymorphisms on the risk of BM, we defined the genotypes with increased risk of BM as “unfavorable” genotypes. These included mLST8:rs26865 with AG/GG genotype and mLST8:rs3160 with TC/CC genotype. Next, we categorized the patients according to the number of risk genotypes. Multivariate Cox proportional hazard analysis showed that the cumulative effect of the risk genotype was “dose dependent,” and as the number of observed risk genotypes increased, the risk of brain metastases also increased significantly. Thus, patients with two unfavorable genotypes exhibited a significantly higher risk for BM (HR = 2.992, 95% CI = 1.695–5.282, *p *< 0.001; Table 3) than those carrying only one unfavorable genotype (HR = 1.945, 95% CI = 0.748–5.052, *p *= 0.172). This cumulative effect of unfavorable genotypes for developing BM was also verified via Kaplan–Meier analyses (*p *= 0.004, Fig. 2d).

**Table 3:** Correlation between different risk genotypes and brain metastasis (combined)

No. of risk genotypes	Patients *N* = 501(%)	Events *N* = 130(%)	Univariate analysis		Multivariate analysis^a^	
			HR (95% CI)	*p*	HR (95% CI)	*p*
0	70 (14.0)	18 (25.7)	1.000		1.000	
1	76 (15.1)	22 (28.9)	1.434 (0.568–3.622)	0.446	1.945 (0.748–5.052)	0.172
2	355 (70.9)	90 (25.4)	2.218 (1.347–3.653)	0.002	2.992 (1.695–5.282)	< 0.001

*HR* hazard ratio, *CI* confidence interval

^a^Multivariate analyses in this table were adjusted for age, sex, smoking status, Karnofsky Performance Status, Body mass index, tumor histology, TNM stage

### Relationship between clinical features and risk SNPs in BM

**Fig. 3:**
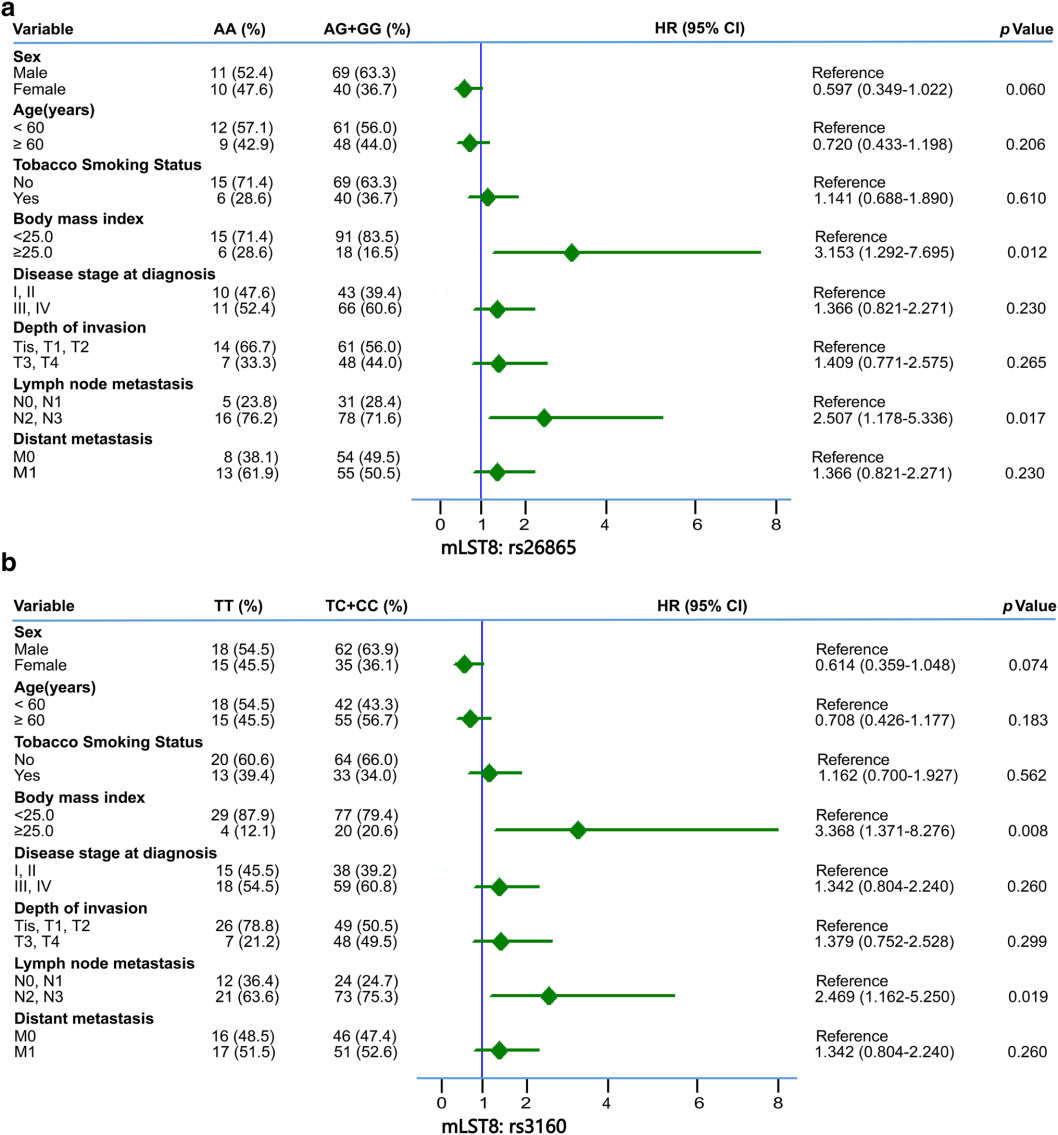
Forest plot of association between variable clinical characteristics and different genotypes of **a** mLST8:rs26865 and **b** mLST8:rs3160. The hazard ratio (HR) with their 95% CI was estimated via Cox proportional hazard analysis

To clarify the relationship between clinical features and the two at-risk SNPs (i.e., mLST8:rs26865 and mLST8:rs3160) in BM of NSCLC, we performed Cox proportional hazard analysis. As is shown in Fig. 3, BM patients with N2/3 stage lymph node metastasis and BMI ≥ 25 kg/m^2^ had a significantly higher tendency of having an AG/GG genotype of mLST8:rs26865, which is an at-risk SNP for BM (HR = 3.153, 95% CI = 1.292–7.695, *p *= 0.012 and HR = 2.507, 95% CI = 1.178–5.336, *p *= 0.016, respectively, Fig. 3a). Similar results were noted for N stage patients with mLST8:rs3160 with TC/CC genotype (HR = 3.368, 95% CI = 1.371–8.276, *p *= 0.008 and HR = 2.469, 95% CI = 1.162–5.250, *p *= 0.019, respectively, Fig. 3b). However, other clinical characteristics, such as age, sex, smoking status, tumor histology, disease stage, T stage, and M stage were not significantly associated with the two at-risk SNPs of BM.

### Correlation of risk SNPs and serum tumor markers in BM

**Fig. 4:**
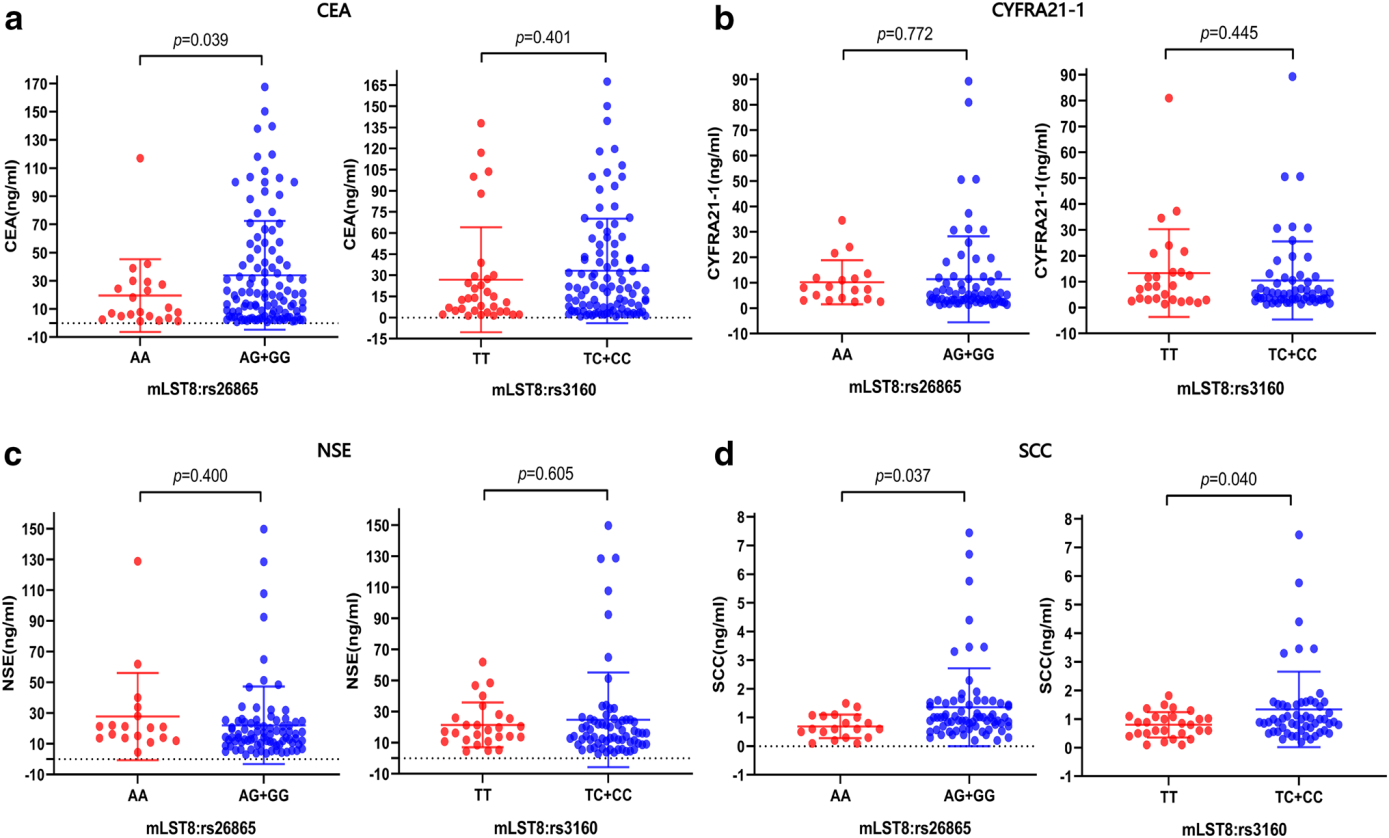
Analysis of the relationship between serum tumor markers and different genotypes of risk SNPs in patients with brain metastasis. The serum tumor markers were **a** CEA, **b** CYFRA21-1, **c** NSE, and **d** SCC antigen

Carcinoembryonic antigen (CEA), cytokeratin fragment 19 (CYFRA21-1), neuron-specific enolase (NSE), and SCC antigen are among the most well-known serum tumor biomarkers for NSCLC, and they have been mainly regarded as predictive or prognostic biomarkers in NSCLC patients. In this study, we investigated the relationship between the two at-risk SNPs and levels of serum tumor markers in BM. The results revealed a significantly higher CEA level in patients with AG/GG genotype of mLST8:rs26865 than in those with AA genotype (mean: 33.90 ng/ml versus 19.51 ng/ml; *p *= 0.039, Fig. 4a). However, there were no differences in CEA level between mLST8:rs3160 with TC/CC genotype and TT genotype (*p *= 0.401), although it tended to be higher in the TC/CC genotype. Moreover, patients with AG/GG genotype of mLST8:rs26865 and TC/CC genotype of mLST8:rs3160 both had a higher CEA level than those with AA genotype (*p *= 0.037, Fig. 4d) and TT genotype (*p *= 0.040). However, neither CYFRA21-1 level nor NSE level was associated with the two at-risk SNPs (i.e., mLST8:rs26865 and mLST8:rs3160) (Fig. 4b, c).

### Association of Ki-67 proliferation index, gene mutations, and risk polymorphisms in BM patients

**Fig. 5:**
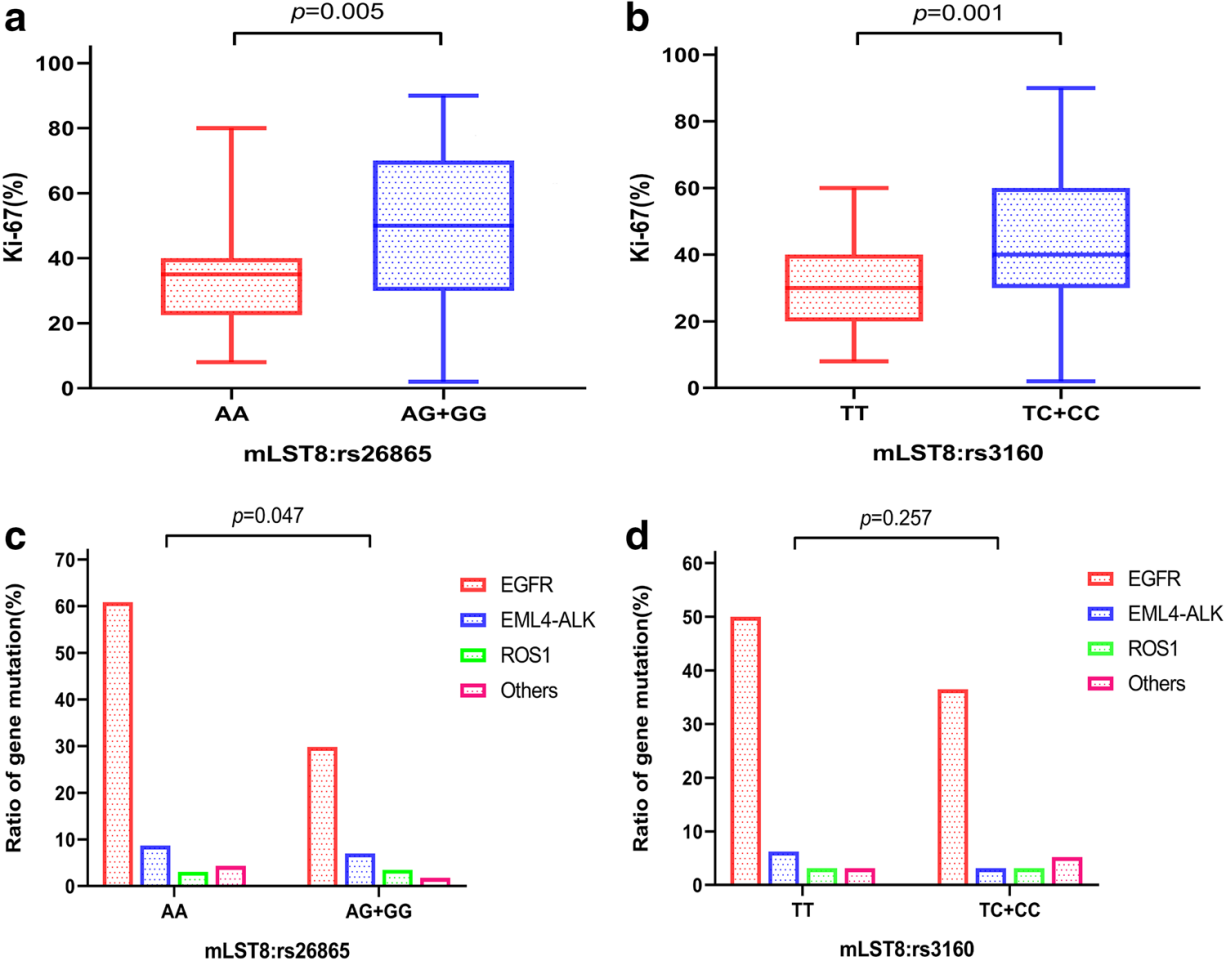
Ki-67 proliferation index and gene mutation frequencies detected in BM patients with at-risk polymorphisms. **a**, **b** Analysis of the association between Ki-67 proliferation index and risk SNPs including mLST8:rs26865 and mLST8:rs3160. **c**, **d** Distribution of the different gene mutation frequency in patients with different risk genotypes

To determine the association between tumor proliferation level and at-risk SNPs, we collected data on the Ki-67 proliferation index. As is shown in Fig. 5a, b, the Ki-67 proliferation index was significantly higher in BM patients with AG/GG genotype of mLST8:rs26865 and TC/CC genotype of mLST8:rs3160 than in those with AA genotype and TT genotype (*p *= 0.005 and *p *= 0.001, respectively). The mean Ki-67 proliferation index of patients with AG/GG genotype of mLST8:rs26865 was 48.17%, while that with AA genotype was 34.40%. Meanwhile, the mean Ki-67 proliferation indices in patients with TC/CC genotype and TT genotype of mLST8:rs3160 were 44.03 and 29.43, respectively (data not shown). These data indicated that the Ki-67 index is higher in BM patients with at-risk genotypes.

Moreover, we attempted to estimate the differences between gene mutations in various SNP genotypes. According to the type of gene, we divided the gene mutations into four groups, namely, EGFR, EML-ALK, ROS1, and others. The results revealed that patients with AA genotype of mLST8:rs26865 had higher gene mutation frequency than those with AG/GG genotype(*p *= 0.047, Fig. 5c). The overall EGFR mutation frequency in the population was 36.9% (48/130), while, it was 61.9% (13/21) in patients with AA genotype of mLST8:rs26865. All the gene mutation frequencies were significantly different between AA and AG/GG genotype of mLST8:rs26865. However, no significant differences were observed between TC/CC genotype and TT genotype of mLST8:rs3160 for gene mutations (*p *= 0.257, Fig. 5d).

### Effect of risk genotypes of SNPs on patient outcome

**Fig. 6:**
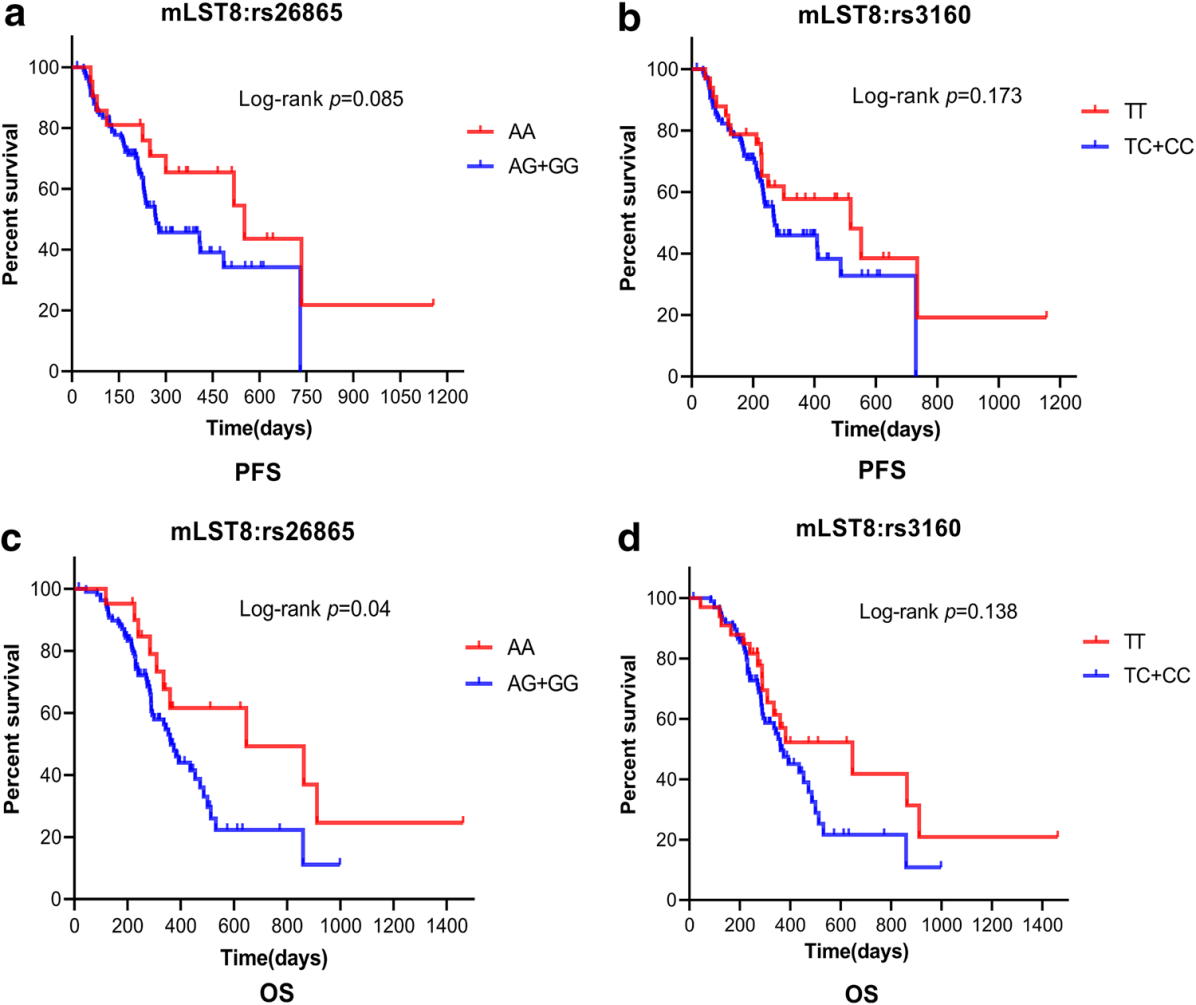
Kaplan–Meier curves indicating the PFS and OS of BM patients with or without unfavorable genotypes. (**a**, **b**) PFS of BM patients with SNPs of mLST8:rs26865 and mLST8:rs3160. (**c**, **d**) OS of BM patients with different genotypes of risk SNPs

Finally, we attempted to determine the effect of risk genotypes of SNPs on overall survival (OS) and progression-free survival (PFS). During the 24-month follow-up period, a total of 81 BM patients (62.3%) died and eight patients (6.2%) were lost to follow-up before 24 months. With respect to median PFS, patients with AG/GG genotype of mLST8:rs26865 tended to have poorer median PFS than those with AA genotype (9.0 months versus 18.4 months), but the difference was not significant (*p *= 0.085, Fig. 6a). Similar results were obtained in mLST8:rs3160, that is, the median PFS of TC/CC genotype was lower than those with TT genotype (9.0 months versus 17.3 months, *p *= 0.173, Fig. 6b). With respect to OS, patients with AG/GG genotype of mLST8:rs26865 had significantly lower median OS than those with AA genotype (12.1 months versus 21.6 months, *p *= 0.04, Fig. 6c). However, there was no statistical difference in the median OS between those with TC/CC genotype and TT genotype of mLST8:rs3160 (*p *= 0.138, Fig. 6d).

## Discussion

BM causes serious neurological symptoms and is the leading cause of mortality in NSCLC. Despite this significant impact, the mechanisms by which BM develops remain unclear, and predictive biomarkers for BM are yet to be identified to date. In this study, we attempted to identify the molecular markers of BM by analyzing the association between eight potential functional polymorphisms of three genes in the mTORC1 pathways and the risk of BM. We found that patients with mLST8:rs26865 and mLST8:rs3160 variant genotypes had an evidently increased risk of BM. To our best knowledge, this is the first study to report that SNPs of mTORC1 pathway-related genes are associated with BM in NSCLC.

In this study, two SNPs associated with BM risk were located in mLST8, which mapped to chromosome 16p13.3 and was composed of 11 exons. mLST8 binds to the catalytic domain of mTOR and is among the crucial component proteins of the mTORC1 pathway (Yang et al. 2013). Furthermore, mLST8 not only enhances the stability of the interaction between RPTOR and mTOR, but also plays a vital role in stimulating mTOR kinase activity (Kakumoto et al. 2015). Therefore, mLST8 is crucial for the regulation of the mTOR pathway. Previous studies have revealed that mLST8 was upregulated in human colon and prostate cancer cells (Sapam et al. 2018) and contributed to tumor progression by activating the mTORC1 complex and its effector protein including translation initiation factor 4E-binding protein 1 (4E-BP1) (Yang and Guan. 2007). mLST8 upregulation could promote phosphorylation of 4E-BP1, which is known to play a role in tumor progression in various cancers including breast, ovarian, and prostate cancers (Armengol et al. 2007). Thus, we hypothesized that mLST8 is essential for tumor progression. A recent study focused on the relationship between SNPs of mLST8 and gastric cancer showed that although mLST8:rs26865 was not statistically related to gastric cancer, its hazard ratio of > 1 suggests a tendency to promote gastric cancer (Xue et al. 2018). These results suggest that our finding of increased BM risk in patients with polymorphisms of mLST8:rs26865 and mLST8:rs3160, at least in part, may be biologically reasonable.

He et al. evaluated the association between SNPs within mTORC1 genes and risk of GC in 1125 patients and 1196 healthy controls in a Chinese population, and the results showed that RPTOR rs1062935 and rs3751934 polymorphisms were not associated with the risk of GC (He et al. 2013). However, in our study, univariate Cox proportional hazard analysis showed that patients with TC/CC genotype of RPTOR:rs1062935 had significant lower risk of BM. These two inconsistent results may be explained by the difference in cancer types, sample size, and genetic susceptibility. In addition, it suggests that larger studies are needed to validate our findings.

Single SNP are well known to have relatively weak predictive power in cellular signaling pathways, while this is greatly enhanced when multiple SNPS are combined (Li et al. 2013a). When we combined two risk SNPs of mLST8:rs26865 and mLST8:rs3160, the cumulative effect of the risk genotype was “dose dependent,” that is, as the number of observed risk genotypes increased, the risk of brain metastases also increased significantly. These results suggest that multiple polymorphisms within the mTORC1 pathway have a cumulative effect and may further enhance the detective power. Nevertheless, evidence for real interactions of the risk SNPs is weak, and the mechanisms behind these high interactions are yet to be elucidated.

Approximately, 45% of patients with NSCLC have lymph node-positive disease, and higher lymph node stage was associated with the risk of BM (Waqar et al. 2018). Moreover, some studies reported that each component of the TNM staging system is correlated with the risk of BM (Mujoomdar et al. 2007). In our study, we analyzed the correlation between clinical status of BM patients and unfavorable genotypes. The results confirmed that BM patients with lymph node metastasis (N2/3) tended to have polymorphisms of rs26865 and rs3160 in mLST8. These observations further indicate that SNPs of mLST8 may be biologically feasible as a marker of BM.

Serum tumor markers remain a simple and economical tool for diagnosing and predicting the prognosis of lung cancer patients. Many serum components such as CEA, CYFRA 21-1, SCC, and NSE have been investigated and considered to be markers of this malignancy (Mauro et al. 2019). With respect to the relationship of serum tumor markers and BM, previous studies have demonstrated that patients with high CEA level had a higher risk of BM and poor prognosis (Lee et al. 2012). In the present study, we confirmed that AG/GG genotype of mLST8:rs26865 was associated with high CEA level and that mLST8 SNPs rs26865 and rs3160 polymorphic variants were associated to high SCC antigen level. Meanwhile, we did not find any association between NSE and the risk of BM, and this may be because NSE is a glycolytic enzyme that is commonly expressed in neuroendocrine tumors, particularly SCLC, rather than NSCLC. Nevertheless, these findings suggested that rs26865 and rs3160 of mLST8 SNPs may play a role in the pathogenesis of BM.

To our best knowledge, Ki-67 is expressed at multiple stages of the cell cycle except in the G0 phase, and the Ki-67 proliferation index is among the most common and widely applied biomarkers of cell proliferation. A previous study has shown that Ki-67 expression is higher in human lung adenocarcinoma tissues than normal lung tissue, and a higher Ki-67 proliferation index was related to the degree of differentiation, TNM stage, lymph node metastasis, and poor OS (Li et al. 2018). Moreover, high Ki-67 proliferation index in BM was associated with unfavorable survival (Berghoff et al. 2014). Our study found that BM patients with AG/GG genotype of mLST8:rs26865 and TC/CC genotype of mLST8:rs3160 expressed a significantly higher Ki-67 index than those with AA genotype and TT genotype. This suggests that patients with mLST8 SNPs rs26865 or rs3160 polymorphic variants may have shorter OS. Coincidentally, by analyzing the effect of risk genotypes of SNPs on BM patient outcomes, we found that patients with AG/GG genotype of mLST8:rs26865 had significantly poorer OS. However, the OS was not significantly different between those with TC/CC genotype and those with TT genotype of mLST8:rs3160. These results indicated that the role of rs26865 and rs3160 polymorphic variants of mLST8 in the development of BM should be further studied.

Recently, one study profiled the characteristics of brain metastases from NSCLC and showed that the EGFR mutation rate in BM was 36.3% (Ferguson et al. 2018). Furthermore, while NSCLC patients with EGFR mutations had a higher risk of brain metastases, they had better prognosis than those with EGFR wild-type mutations (Hsu et al. 2016). In our study, the frequency of EGFR mutation was 36.9% in 130 BM patients, which is consistent with previous finding (Ferguson et al. 2018). Moreover, the frequency of EGFR mutation was significantly higher in patients with AA genotype of mLST8:rs26865 than those with AG/GG. This may indicate that patients with wild-type genotype of mLST8:rs26865 SNP, at least in part, may have better survival. Nevertheless, we did not detect other gene mutations such as TP53 and K-ras that have been previously confirmed to have a high frequency of mutations in brain metastases (Ma et al. 2018). This is due to the limitations of patient genetic testing data and underline that we should collect more complete information to better investigate the association between polymorphisms in mTORC1 pathway and BM of NSCLC.

This study has some limitations that should be considered when determining the generalizability of the results. One limitation is the relatively small sample size, and thus and this may have influence the finding of weak effect of polymorphisms in BM. Furthermore, we selected potential functional genetic variants instead of tagging SNPs of mTORC1 pathway in the analysis, and thus some other important genetic variants may have not been included. Finally, the functional mechanisms by which potential genetic variations affected BM have not yet been elucidated. Therefore, additional larger, well-designed, and in-depth studies should be performed to overcome these limitations.

In conclusion, polymorphisms of mTORC1 pathway were associated with the risk of BM. We found that rs26865 and rs3160 polymorphic variants of mLST8 were significantly associated with the risk of BM, particularly among those with lymph node metastasis (N2/3), high CEA and SCC antigen level, and high Ki-67 proliferation index and gene mutation frequency. These findings show that SNPs, combined with other predictive indicators, may be valuable biomarkers for identifying NSCLC patients with a high risk of brain metastasis.

## Electronic supplementary material

Below is the link to the electronic supplementary material.

Supplementary material 1 (DOCX 15 kb)

Supplementary material 2 (DOCX 16 kb)
