# Supplementary material for: Effects of single-nucleotide polymorphisms in the mTORC1 pathway on the risk of brain metastasis in patients with non-small cell lung cancer
Source: J Cancer Res Clin Oncol. 2019 Oct 22;146(1):273–85. doi: 10.1007/s00432-019-03059-y (PMC6942024; doi:10.1007/s00432-019-03059-y)
Supplement: Supplementary file 1 — Supplementary material 1 (DOCX 15 kb) [file 432_2019_3059_MOESM1_ESM.docx]

**Supplementary Table S1.** Primer sequences for 8 SNPs in genotyping.

| SNP-ID | Forward primer | Reverse primer |
| --- | --- | --- |
| mTOR:rs1883965 | ACGTTGGATGCTGGATCTCTGAGAGGTGTC | ACGTTGGATGATTGTGTATCTCTGCCCCAG |
| mTOR:rs2536 | ACGTTGGATGTGGTGTCTAGACATGGCTAC | ACGTTGGATGTGCTGAACACAGGGAAGGTC |
| mLST8:rs26865 | ACGTTGGATGAGAGCTAGCAGTCATCAGCC | ACGTTGGATGTGTCCCCGACATTTAGTCTC |
| mLST8:rs3160 | ACGTTGGATGTGGTGGTGTTCTCTATGGAC | ACGTTGGATGAAAGCTGCTGAGGGGTCTGA |
| RPTOR:rs1062935 | ACGTTGGATGTTCCCATCCAGTCCTTCAAC | ACGTTGGATGTGCCTTTTGACAGGTGGTGA |
| RPTOR:rs12602885 | ACGTTGGATGTGGAAACGTACAGCCTCAG | ACGTTGGATGAAGAAGCTCCGCAGACACAC |
| RPTOR:rs3751932 | ACGTTGGATGAGAAGATGCTCTTGGCCACG | ACGTTGGATGTGGCTTCATTCTGGCGGTGA |
| RPTOR:rs3751934 | ACGTTGGATGTCGCTCGCTCTTGGCCTGAT | ACGTTGGATGTCAGAGCATTAGCTGCAG AA |
