# Supplementary material for: Effects of single-nucleotide polymorphisms in the mTORC1 pathway on the risk of brain metastasis in patients with non-small cell lung cancer
Source: J Cancer Res Clin Oncol. 2019 Oct 22;146(1):273–85. doi: 10.1007/s00432-019-03059-y (PMC6942024; doi:10.1007/s00432-019-03059-y)
Supplement: Supplementary file 2 — Supplementary material 2 (DOCX 16 kb) [file 432_2019_3059_MOESM2_ESM.docx]

**Supplementary Table S2.** Genes and SNPs selected for analysis.

| Gene | SNP | Allelic change | SNP position | MAF^a^ | Function prediction |
| --- | --- | --- | --- | --- | --- |
| mTOR | rs1883965 | G>A | Exon | 0.10 | TFBS^c^ |
|  | rs2536 | T>C | 3’UTR^b^ | 0.07 | miRNA [binding](javascript:;) [site](javascript:;) |
| mLST8 | rs3160 | T>C | 3’UTR | 0.47 | miRNA [binding](javascript:;) [site](javascript:;) |
|  | rs26865 | A>G | near 5’ | 0.48 | TFBS |
| RPTOR | rs3751934 | C>A | 3’UTR | 0.36 | TFBS |
|  | rs1062935 | T>C | 3’UTR | 0.46 | TFBS |
|  | rs3751932 | T>C | 3’UTR | 0.13 | TFBS |
|  | rs12602885 | G>A | 5’UTR | 0.31 | TFBS |

^a^MAF: Minor Allele Frequency; ^b^UTR: [Untranslated](javascript:;) [Region](javascript:;); ^c^TFBS: Transcription Factor Binding Site.
